# Effects of Laboratory Housing on Exploratory Behaviour, Novelty Discrimination and Spatial Reference Memory in a Subterranean, Solitary Rodent, the Cape Mole-Rat (*Georychus capensis*)

**DOI:** 10.1371/journal.pone.0075863

**Published:** 2013-09-11

**Authors:** Maria Kathleen Oosthuizen, Anne-Gita Scheibler, Nigel Charles Bennett, Irmgard Amrein

**Affiliations:** 1 Department of Zoology and Entomology, University of Pretoria, Pretoria, South Africa; 2 Department of Anatomy, University of Zurich, Zürich, Switzerland; University of Queensland, Australia

## Abstract

A large number of laboratory and field based studies are being carried out on mole-rats, both in our research group and others. Several studies have highlighted the development of adverse behaviours in laboratory animals and have emphasised the importance of enrichment for captive animals. Hence we were interested in evaluating how laboratory housing would affect behavioural performance in mole-rats. We investigated exploratory behaviour, the ability to discriminate between novel and familiar environments and reference memory in the solitary Cape mole-rat ( *Georychus* *capensis* ). Our data showed that both wild and captive animals readily explore open spaces and tunnels. Wild animals were however more active than their captive counterparts. In the Y maze two trial discrimination task, wild animals failed to discriminate between novel and familiar environments, while laboratory housed mole-rats showed preferential spatial discrimination in terms of the length of time spent in the novel arm. The performance of the laboratory and wild animals were similar when tested for reference memory in the Y maze, both groups showed a significant improvement compared to the first day, from the 3rd day onwards. Wild animals made more mistakes whereas laboratory animals were slower in completing the task. The difference in performance between wild and laboratory animals in the Y-maze may be as a result of the lower activity of the laboratory animals. Laboratory maintained Cape mole-rats show classic behaviours resulting from a lack of stimulation such as reduced activity and increased aggression. However, they do display an improved novelty discrimination compared to the wild animals. Slower locomotion rate of the laboratory animals may increase the integration time of stimuli, hence result in a more thorough inspection of the surroundings. Unlike the captive animals, wild animals show flexibility in their responses to unpredictable events, which is an important requirement under natural living conditions.

## Introduction

Exploratory behaviour has important survival implications for wild animals. Once animals have entered a novel environment, they need to familiarize themselves with their environment in order to establish home ranges, efficiently utilize resources and effectively avoid predators [1]. The primary method of gathering information about their spatial surroundings is by exploratory behaviour, hence creating a spatial representation of their environments [2]. In order to gain new information about their surroundings, animals are faced with two opposing goals, investigating for possible threats while at the same time remaining as inconspicuous as possible [3]. The balance between risk taking and the potential gain of resources varies with different situations and on the animal’s motivation and emotional state [4].

Laboratory animals are habitually maintained in an environment that is far less complex compared to their natural habitat. The complexity of an environment is inversely related to its predictability, therefore laboratory animals commonly inhabit a fairly predictable habitat [5]. Potential for exploration and species specific behaviours is limited for laboratory animals, decreasing the overall variability in behaviour. Captive animals frequently develop various forms of adverse behaviours caused by stress and boredom [6]. Stress can cause anxiety behaviour in mammals, which may be presented as escape or avoidance, aggression (that includes anger, clawing and biting), freezing or immobility (in order to assess risks and remain concealed) and submissive behaviour [7]. Stress can have complex effects on cognition and the specific effects vary depending on the nature of the stress as well as subject specific characteristics. Both recognition and spatial memory may be affected by stress, in both cases, acute stress prior to learning or retrieval impairs memory [8]. In addition, the effects of stress on recognition memory are also influenced by sex and arousal level of the test subjects [9,10]. In contrast, brief periods of stress immediately prior to learning may actually enhance spatial memory [8].

African mole-rats (family Bathyergidae) are subterranean rodent moles endemic to sub-Saharan Africa. The Cape mole-rat ( *Georychus* *capensis* ) is a solitary species that inhabits moderately moist (mesic) environments in the south western parts of South Africa [11]. They inhabit sealed but dynamic burrow systems and rarely emerge aboveground. Tunnels are extended or altered whenever the soil is soft enough, thus mostly after rains, in search of food sources. Mole-rats are both physically and physiologically well adapted for life underground [11]. Physical characteristics include short legs, a streamlined body shape, and sparsely distributed sensory hair over their bodies that by means of mechanosensory stimuli aid orientation [12]. Since mole-rats spend their entire life underground their visual system is regressed. Although morphologically normal, their eyes are microphthalmic and the projections to all CNS visual structures as well as the structures themselves are reduced to varying degrees. Current data suggests that mole-rats have low visual acuity and only a crude capability to discriminate brightness and form, and their vision clearly is not suited for aboveground orientation [13]. Thus, in order to navigate their tunnel systems mole-rats rely on tactile stimuli and memory [14].

African mole-rats have been the subject of numerous studies by our research group for several decades. Extensive studies have been carried out not only in the field but also in the laboratory, therefore we were interested in evaluating the effect of housing conditions on behavioural performance between laboratory and wild captured animals. This was achieved by comparing two groups of animals, one group was freshly captured and tested within two weeks (wild group), whereas the other group was maintained in the laboratory in a simple environment without enrichment for at least one year (laboratory group). We tested and compared the general exploratory behaviour, the ability to recognise spatial novelty and the proficiency to build reference memory in consecutive trials over four days.

## Materials and Methods

Experimental animals were captured in during two field trips using Hickman live traps [15]. Female Cape mole-rats were collected near Darling, Western Cape, South Africa (33^°^22’ S, 15^°^25’ E). Animals were transported to the University of Pretoria where experiments were conducted.

Two groups of animals were tested, the laboratory group consisted of ten female Cape mole-rats (body weight 166±42g) that were trapped and maintained in captivity for at least one year prior to the commencement of experiments. Nine female Cape mole-rats comprised the wild group (body weight 137±31g), they were subjected to experimental testing immediately after arrival in the laboratory (within two weeks of capture). While in the laboratory, both groups of animals were maintained individually in plastic crates lined with wood shavings and tissue paper was provided as nesting material. Animals were fed *ad libitum* on chopped sweet potato, apple, gem squash and carrot.

Experimental procedures were approved by the Animal Use and Care Committee at the University of Pretoria (EC013-09). A trapping permit was obtained from the Western Cape Nature conservation authority.

### Apparatus

The apparatus for the first experiment as previously described [16] consisted of an open topped wooden box (dimensions 20x40x20 cm), painted white. From one of the long sides of the box, three black plastic tunnels projected externally (20 cm long, 7 cm diameter). The tunnels were evenly spaced along the side of the box and positioned 1 cm above the floor of the box. The floor of the box was lightly covered with wood shavings. Between animals, the box and tunnels were wiped with 95% alcohol and subsequently with distilled water.

For the second and third experiment the apparatus was a Y-maze constructed of Perspex, each arm measuring 50x10x20cm. Each arm had an escape hole on the distal end 2 cm above the floor with a tunnel attached. Both distal and proximal ends of each arm could be blocked off with a guillotine door.

### Experimental design

#### Experiment 1: Exploration in tunnel maze

The first experiment was designed to test the exploratory behaviour and general activity levels of Cape mole-rats. Animals were placed in the centre of the box and observed for 3 minutes. The latency to enter the first tunnel, total number of entries, total and mean duration spent in tunnels and number of tunnels visited was recorded. All four feet of the animal were required to be inside a tunnel to be considered as an entry.

#### Experiment 2: Response to spatial novelty in Y-maze

The second experiment was a two trial task for discriminating between novelty and familiarity based on a free choice exploration paradigm [17]. During the exposure phase all distal ends of the arms were blocked off as well as the proximal end of one arm. The animal was placed in the distal end of one of the open arms (start arm), facing away from the centre of the maze. The animal was allowed to explore the start arm and the second arm for 5 minutes. The animal was then returned to its home cage for one minute during which the blocked arm (novel arm) was opened. The animal was then placed back in the start arm and allowed to explore all 3 arms of the maze for two minutes. The total time and number of entries into each arm was measured for both the exploratory phase and the test phase.

#### Experiment 3: Reference memory in paddling Y-maze

In this experiment, the bottom of the Y-maze was filled with about 2 cm of water (at room temperature) to motivate animals to move. On the distal end of one arm was an escape hole connected to a Perspex tunnel and a dry nest box. Blocked tunnels were also placed at ‘false exits’ so that all arms looked identical from the centre. Animals were released into the start arm facing away from the centre of the maze and allowed one minute to find the escape hole. The number of wrong entries was recorded as well as the time to find and enter the escape hole. The starting position for animals was alternated randomly between two of the arms of the maze, such that for each day 5 trials started in each of the two start positions. Animals always had to turn right to find the escape hole. Animals were subjected to the paddling Y-maze for four consecutive days consisting of 10 trials per day. For each individual the time between trials varied between 45 and 60 minutes. Animals appeared to get tired or less interested following the 5th trial, which resulted in a noticeable drop in performance. Therefore means for latency and wrong entries were calculated for each day using trials 1-5.

Measurements of body mass prior to and after the experimental phase showed no changes (data not shown), indicating that the tests were not overly stressful for the animals.

### Data acquisition and statistics

All experiments were recorded with an overhead video recorder and analysed manually after the completion of the experiments. Statistical analyses were performed with IMB SPSS Statistics version 20. Comparisons between wild and laboratory animals of experiment 1 and 2 were assessed using multivariate General Linear Model (GLM) with behavioural data as independent and housing conditions as fixed factors. General linear model (GLM) repeated measures were employed for the analysis of experiment 3. Significance level was set at 0.05 and the main effects were tested with LSD.

## Results

### Experiment 1: Exploration in tunnel maze

**Figure 1:**
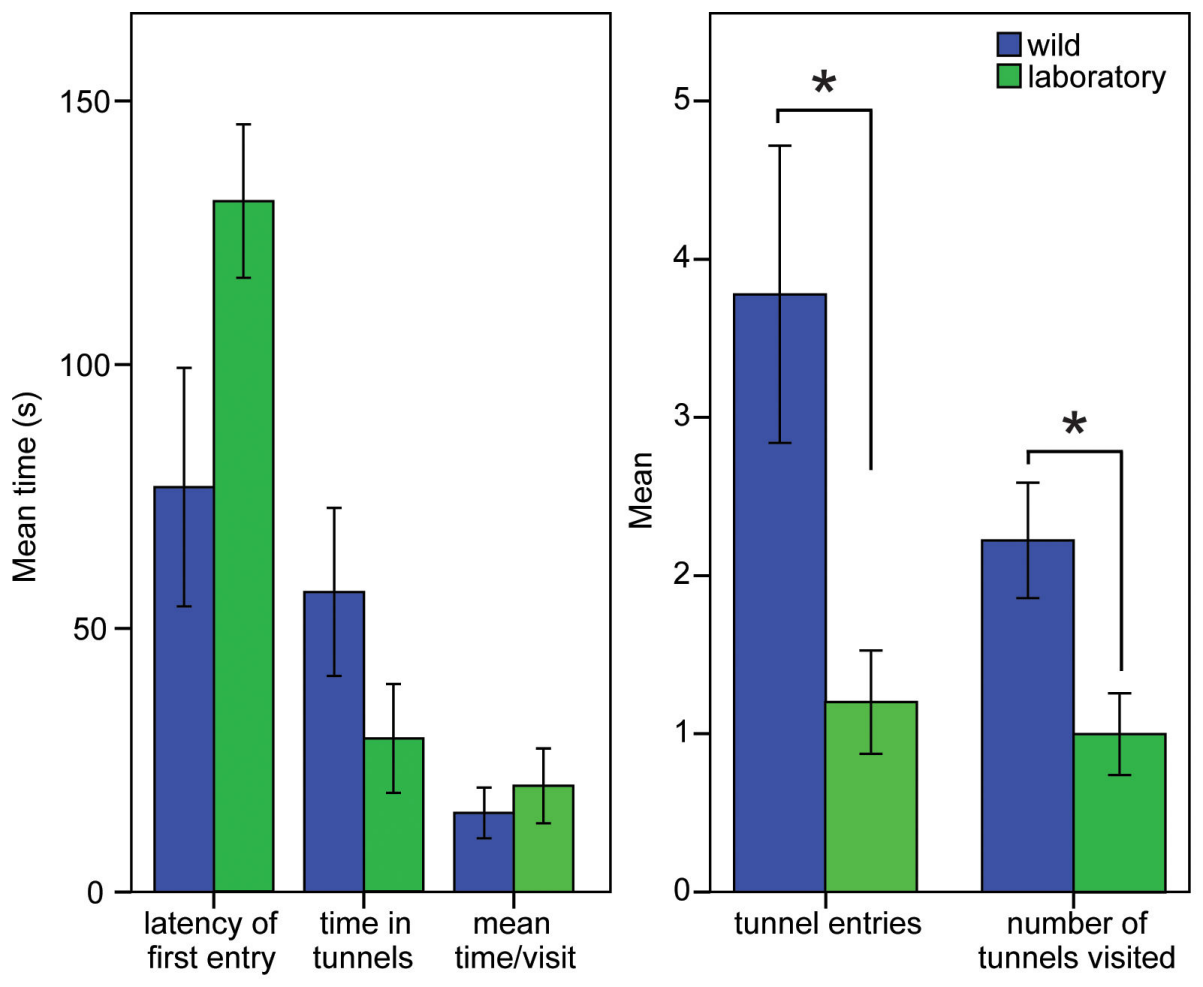
A. Time related exploration measurements in the tunnel maze for the Cape mole-rat. The latency to enter the first tunnel was longer for laboratory animals, however the difference was not significant. The total time spent in the tunnels and the mean time per visit revealed no differences. B. Activity measures shows that wild Cape mole-rats were more active than their laboratory counterparts, both in terms of tunnel entries and the number of tunnels visited (max 3). Significance is marked with *, the exact p-values are specified in the results section. Bars indicate SE.

Wild trapped Cape mole-rats made significantly more entries into tunnels (p=0.027) and they visited a larger number of different tunnels (p=0.013) than laboratory-housed Cape mole-rats. No difference was evident between the wild trapped and laboratory housed mole-rats in terms of latency to enter the first tunnel, total time spent in tunnels or the mean time spent in each tunnel (Table 1; Figure 1).

**Table 1:** Comparison between wild and lab mole-rats.

Expt	Parameter	wild (n=9)	lab (n = 10)	P	
Expt 1	Latency	76.78 ± 67.83	130.9 ± 46.07	p=0.056	
	Time in tunnels	56.89 ± 47.85	29 ± 32.59	p=0.152	
	Mean time/entry	14.97 ± 14.42	20.13 ± 22.45	p=0.564	
	Tunnel entries	3.78 ± 2.82	1.2 ± 1.03	p=0.027	
	Number of tunnels	2.22 ± 0.816	1 ± 0.26	p=0.013	
Expt 2	Exploration phase				
	Time in start arm	156.57 ± 23.65	162.4 ± 29.02	p = 0.645	
	Time in 2nd arm	143.22 ± 23.64	137.6 ± 29.02	p = 0.652	
	Entries in start arm	9.67 ± 8.19	4.2 ± 2.04	p = 0.083	
	Entries in 2nd arm	10.11 ± 8.43	4.9 ± 2.08	p = 0.075	
	Test phase				
	Time in start arm	32 ± 21.84	20.6 ± 14.68	p = 0.195	
	Time in 2nd arm	40.11 ±15.07	31.4 ± 23.31	p = 0.353	
	Time in novel arm	47.89 ±21.47	68 ± 15.99	p = 0.032	
	Entries in start arm	5 ± 4.09	1.7 ± 1.16	p = 0.044	
	Entries in 2nd arm	5.89 ± 3.93	2 ± 0.94	p = 0.018	
	Entries in novel arm	4.33 ± 1.8	2.6 ± 1.08	p = 0.02	

### Experiment 2: Response to Spatial Novelty in Y-Maze

**Figure 2:**
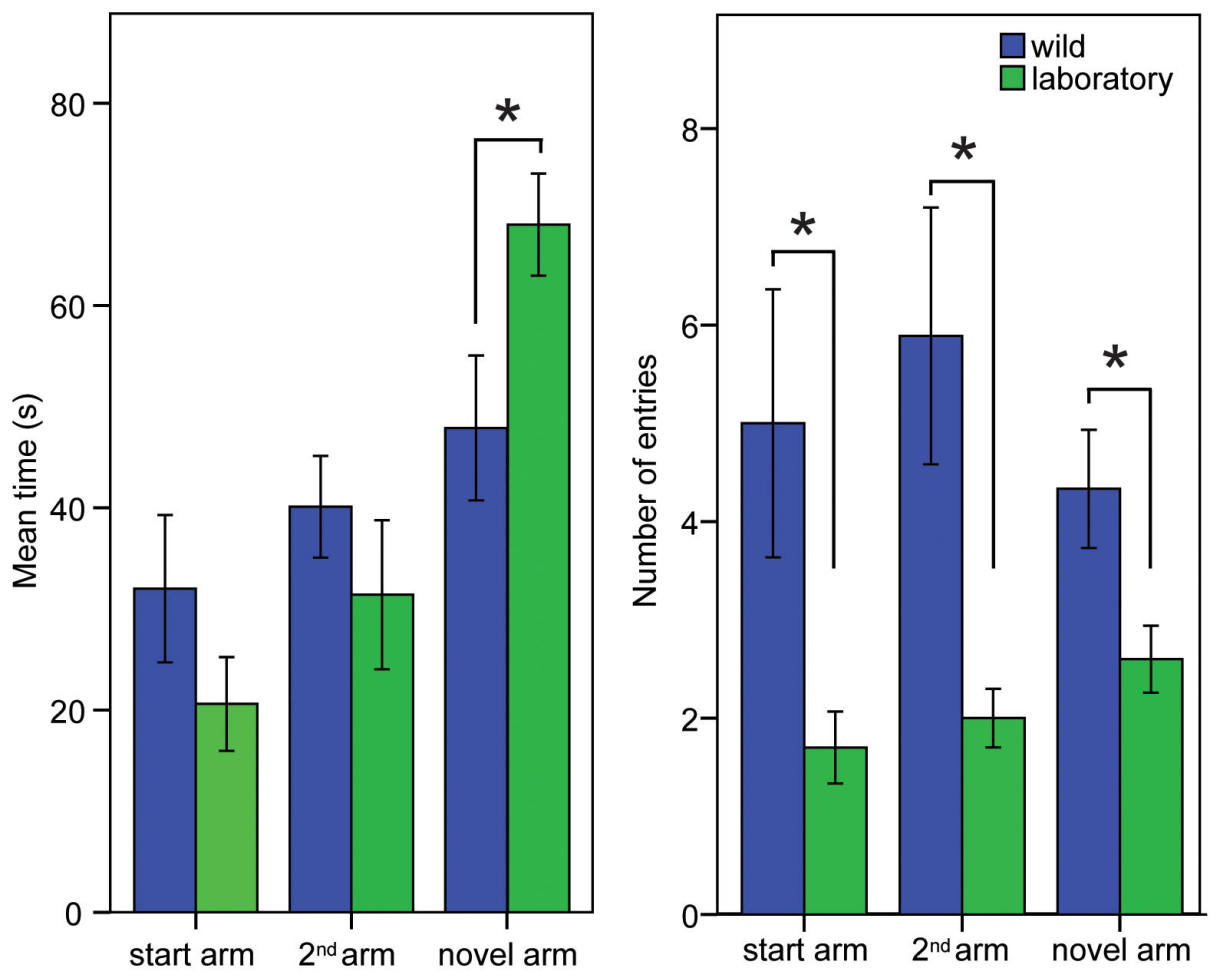
A. Time measurements for spatial novelty in the Y-maze for the Cape mole-rat. During the test phase, laboratory maintained Cape mole-rats showed a strong preference to the novel arm compared to their wild counterparts. B. Activity measures show that during the test phase, wild Cape mole-rats enter all three arms of the maze more often than laboratory Cape mole-rat. Significance is marked with *, the exact p-values are specified in the results section. Bars indicate SE.

In the exposure phase, wild and laboratory mole-rats did not perform differently with respect to time spent in either of the two initial arms of the maze, or the number of entries into each of the arms (Table 1). In the test phase, visits of laboratory Cape mole-rats in the novel arm were significantly longer than those of wild animals (p=0.032). The Cape mole-rats of the wild population were significantly more active than the laboratory animals entering all arms more often (start arm: p=0.044; 2^nd^ arm: p=0.018; novel arm: p=0.02) (Table 1, Figure 2).

### Experiment 3: Reference memory in paddling Y-maze

**Figure 3:**
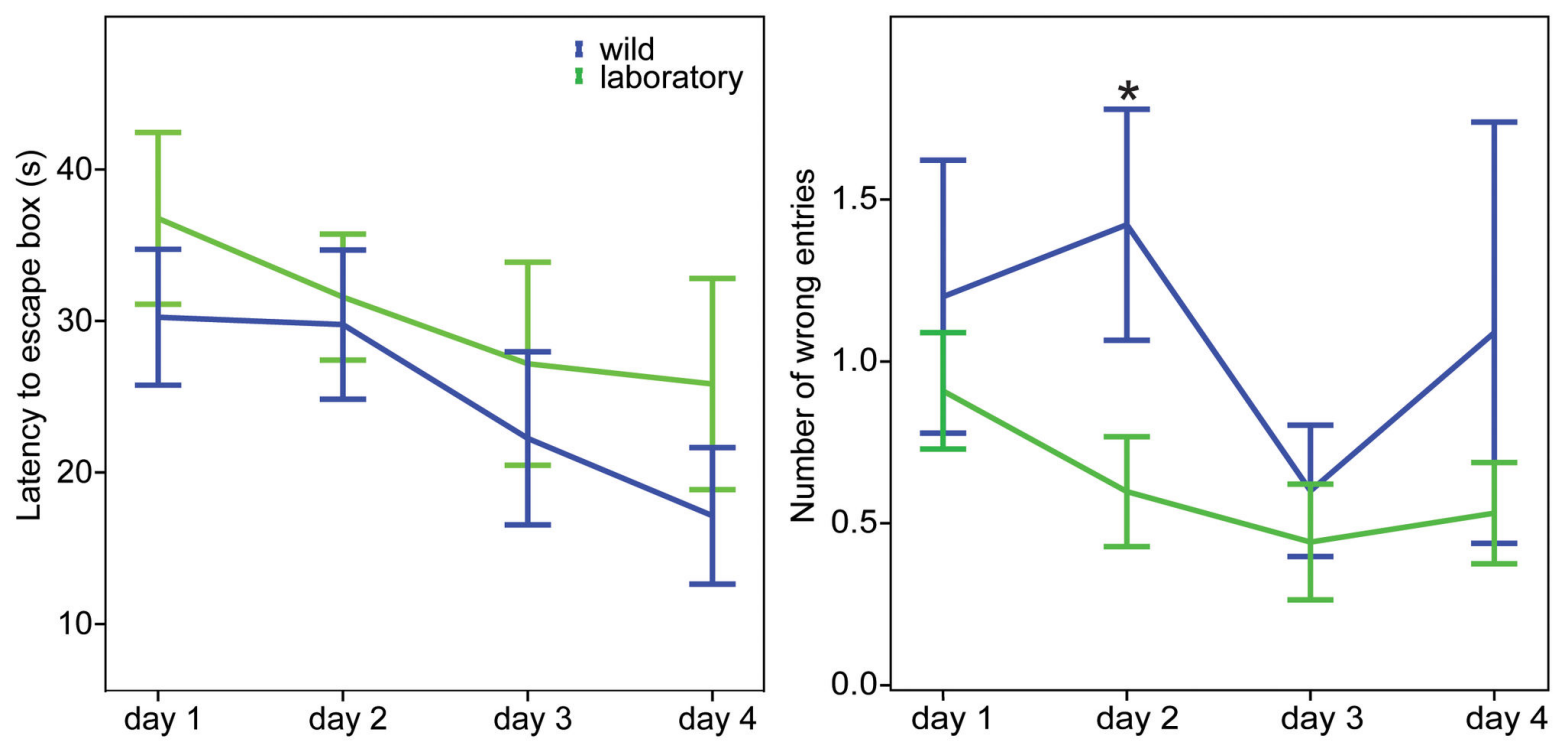
A. Escape latency in the paddling Y-maze shows that wild and laboratory Cape mole-rats found the escape hole significantly faster on the 3^rd^ day compared to the 1^st^ day. By day 4, all animals reached the escape hole significantly faster than on day 1. B. Incorrect entries into blocked arms of wild Cape mole-rats are higher on the 2^nd^ day than for the laboratory animals on day 2 (*) Significance is marked with, the p-values are specified in the results section, and bars indicate SE, d1-4 refers to experimental day 1 to day 4.

For both the laboratory and wild Cape mole-rats, the latency to enter the escape hole decreased significantly from the first day to day 3 (laboratory: p=0.018, wild: p=0.005). The performance comparison between days one and four demonstrated a significant improvement in the ability to find the escape hole (laboratory: p<0.001; wild: p=0.002, Figure 3). The number of wrong entries made differed only on day 2, when wild animals made more errors than laboratory animals (p=0.01).

## Discussion

### Exploratory behaviour

Mole-rats spend their entire lives in self-constructed tunnel systems with a diameter slightly larger than their bodies. The study animals were therefore predicted to be more reluctant to explore the open space and spend the majority of their time in the tunnels. However, both captive and wild mole-rats readily explored the open space as well as the different tunnels. The observed behaviour is consistent with previous reports from mole-rats. Deacon et al. (2012) compared naked mole-rats to C57BL/6 mice in a similar tunnel maze and the performance of the mole-rats appeared comparable to the Cape mole-rats in our study. Compared to laboratory mice, mole-rats are less active and slower in exploration [16]. It has been demonstrated that captive blind mole-rats initially explore slowly until they are more familiar with the environment [18]. The lower activity and slower exploration of tunnels by mole-rats may be a characteristic of subterranean mammals with very low visual acuity.

Although laboratory housed Cape mole-rats explored both the open space and the tunnels in the tunnel maze, they were much less active compared to their wild counterparts. Similarly, laboratory guinea pigs were also found to be less exploratory compared to their wild counterparts [19]. Wild animals are generally considered as more exploratory and responsive to their environment than laboratory animals [20], while some domesticated animals show a reduction in emotional reactivity [21].

Wild Cape mole-rats exhibited more variability in their behaviour than the laboratory conspecifics. A relationship between activity and anxiousness was observed in laboratory opossums, more active animals were less anxious, and likewise, more active animals showed more variable behaviour [22]. As a result of the more variable behaviour of the wild animals, differences in some of the behavioural measurements of the two groups appeared non-significant. The differences in activity between wild and laboratory housed Cape mole-rats may be due to a shift from exploratory behaviour in the wild animals to a fearful or aggressive phenotype as has been described for other captive animals [23]

Laboratory maintained Cape mole-rats differed from domesticated laboratory animals by being more stressed and aggressive (backing into a corner, head arched backwards with open mouth making snorting noises and biting into the air) and showing defensive behaviour (freezing). In contrast, laboratory mice showed little risk assessment and took greater risks while wild mice were more reluctant to enter open spaces [3]. Domesticated laboratory guinea pigs were significantly less stressed than their wild counterparts, and showed less aggressive behaviour and became more tolerant towards conspecifics [19]. Domestic animals are the product of a selection process for favourable ‘laboratory’ traits over multiple generations such that they are not overly active, nervous or aggressive [19]. As a result of their highly xenophobic behaviour, Cape mole-rats have not been bred successfully in the laboratory, thus it remains an open question whether they can be domesticated.

### Spatial novelty

Mice appear to rely heavily on visuo-spatial information to orientate and discriminate between novel and familiar environments, strains with a visual defect resulting in severely reduced vision have been shown to exhibit no preferential exploration for novelty [24]. All mole-rat species are microphthalmic, and are therefore unable to make use of visual information for orientation. Indeed we observed strong thigmotaxis in the animals indicating that they heavily depend on mechanosensory-mediated orientation [12]. Captive mole-rats showed a preference for spatial novelty, while the wild mole-rats failed to recognise the novel environment in the Y-maze. Exploration can be subdivided into two categories, namely inspective and inquisitive exploration [25]. Inspective exploration is defined as stimuli available in the animal’s field of ‘view’ while inquisitive exploration defines the stimuli that the animal needs to search for [26]. The duration of the visits in the novel arm presents an index of inspective exploration, while the number of visits to the novel arm signifies inquisitive exploration [24]. According to these criteria, captive Cape mole-rats primarily performed inspective exploration, and wild Cape mole-rats performed indiscriminately inquisitive exploration, similar to their behaviour in the first experiment. Several factors have been identified to influence recognition memory, and therefore exploratory behaviour in the Y-maze. It appears that only inquisitive exploration is conserved in aged rats, as opposed to young rats that perform both inquisitive and inspective exploration [27]. It is improbable that age affected novelty recognition in the mole-rats since all mole-rats were randomly collected from the field, thus ages are unknown for both the wild and captive mole-rats. Wild mole-rats collectively did not exhibit a preference for the novel arm. As a solitary species, each animal is responsible for the construction of its entire tunnel system. When encountering an unfamiliar tunnel in nature, (i.e. one that they have not dug) it may suggest that a conspecific has breached into their tunnel system, and could be perceived as an increased risk of encountering another animal, hence decrease the motivation for exploration. Alternatively, the inquisitive exploration seen in the wild animals may be a form of patrolling to ensure the safety of their environment.

Chronic stress has been shown to impair recognition memory in rats [28], but brief periods of stress immediately prior to spatial memory tests can even affect performance positively [8]. This seems to be the case in the laboratory mole-rats as they did show a preference for the novel arm in the experiment even though they appeared to be more stressed than their wild conspecifics.

A more plausible explanation may be that the slower locomotion of the captive mole-rats may provide them the opportunity to investigate more accurately. The wild animals were more active, moved faster and showed strong thigmotaxis. In addition, they are unable to use visual cues for navigation as a result of their low visual acuity [13]. Therefore they are more likely to miss a novel space when they are not running along the wall with the opening to the novel or unfamiliar arm. Slow exploration may allow laboratory animals a longer integration time of stimuli, hence a more thorough inspection of the surroundings.

### Acquisition of reference memory

Both wild and captive mole-rats showed a significant reduction in time to reach the escape hole over four days, indicating the acquisition of reference memory. The ability of subterranean rodents to build a reference memory has also been demonstrated in the blind mole –rat ( *Spalax* *ehrenbergi* ) where, compared to surface dwelling rodents, they performed comparably. Moreover, when tested over time, the blind mole-rat outperformed the other rodents in terms of memory retention [29].

The complexity of the housing conditions did not affect the learning ability in the Cape mole-rat. Captive mole-rats did not show impairment for spatial learning when compared to the wild mole-rats in either the latency to find the escape hole or number of errors per trial. In fact, wild Cape mole-rats made more errors than the captive ones. Previous studies tested memory of freshly trapped Cape mole-rats in a simplified Rabinovich and Rosvold maze [30] and female Cape mole-rats were found to exhibit more erratic behaviour than males [31].

In contrast to our results, Du Toit and colleagues tested the learning ability of a social mole-rat species, the Natal mole-rat ( *Cryptomys* *hottentotus* *natalensis*), in a complex maze and showed that laboratory maintained animals made significantly more navigation errors and were significantly less likely to complete the maze than their wild trapped counterparts [14].

Wild Cape mole-rats had a slightly lower latency to find the escape holes than the captive animals on all days, although this was not significantly so. However, wild Cape mole-rats made more errors in attaining the goal, although only on the second day this was significant. Even with the higher number of wrong turns, wild mole-rats were still faster in finding the escape hole, implying that they are moving much faster than the captive mole-rats. The higher number of wrong entries of the wild mole-rats may again be a result of the speed at which the animals travel.

## Conclusions

Captive animals are frequently maintained in an impoverished environment with a much reduced complexity, while the predictability is increased compared to the natural environment [5]. In such conditions animals are known to become less active and disinterested in their environment. The lack of stimulation induces boredom that may lead to displacement behaviours. They are not motivated to explore and overreact to any unexpected or new event with aggression and fear [23].

Substantial behavioural differences were observed in laboratory housed Cape mole-rats compared to wild mole-rats. Laboratory mole-rats showed classic behaviours associated with a lack of stimulation such as reduced activity as well as aggressive reactions towards unexpected experiences. However, their performance was more focussed and attentive. Wild animals exhibited higher activity levels and lower levels of anxiety, but their exploration appeared to be driven by searching for salient stimuli. Extended time in the laboratory appears to reduce the speed of risk assessment in captive Cape mole-rats, and they appear to lose flexibility to deal with unpredictable events.
